# Effect of Nanobainite Content on the Dry Sliding Wear Behavior of an Al-Alloyed High Carbon Steel with Nanobainitic Microstructure

**DOI:** 10.3390/ma12101618

**Published:** 2019-05-17

**Authors:** Zhaohuan Song, Songhao Zhao, Tao Jiang, Junjie Sun, Yingjun Wang, Xiliang Zhang, Hongji Liu, Yongning Liu

**Affiliations:** 1 State Key Laboratory of Mechanical Behavior of Materials, Xi’an Jiaotong University, Xi’an 710049, China; songzhaohuan@163.com (Z.S.); junjie.sun1987@163.com (J.S.); wangyingjun0222@163.com (Y.W.); 2 College of Materials Science and Engineering, Hebei University of Engineering, Handan 056038, China; zhaosonghao2019@163.com (S.Z.); xl_zapply@163.com (X.Z.); 3 Central Lab, EBOHR Luxuries International LTD., Shenzhen 518000, China; tedivy@sina.cn

**Keywords:** nanobainite, dry sliding wear, hardness, oxidative wear

## Abstract

In this work, a multiphase microstructure consisting of nanobainte, martensite, undissolved spherical carbide, and retained blocky austenite has been prepared in an Al-alloyed high carbon steel. The effect of the amount of nanobainite on the dry sliding wear behavior of the steel is studied using a pin-on-disc tester with loads ranging from 25–75 N. The results show that, there is no significant differences in specific wear rate (SWR) for samples with various amounts of nanobainite when the normal load is 25 N. While, the SWR firstly decreases and then increases with increasing the amount of nanobainite, and the optimum wear resistance is obtained for samples with 60 vol.% nanobainite, when the applied load increases to 50 and 75 N. The improved wear resistance is attributed to the peak hardness increment resulted from the transformation of retained austenite to martensite, work hardening, along with amorphization and nanocrystallization of the worn surface. In addition, the highest toughness of the samples with 60 vol.% nanobainite is also proven to play a positive role in resisting sliding wear. EDS (energy dispersion spectrum) and XRD (X-ray diffraction) examinations reveal that the predominant failure mechanism is oxidative wear.

## 1. Introduction

It is said that 23% of the world’s total energy is lost due to friction [1], and about 70% of components fail because of wear and/or fatigue [2]. Therefore, developing materials with high wear resistance or low friction coefficient is of critical significance. In recent years, a novel nanobainitic steels have been widely studied for its excellent mechanical properties [3,4,5,6,7,8,9], which made them have significant potential use in manufacturing mechanical parts, such as bearings, gears, as well as defense applications. Among these studies, the primary focus is evaluating the wear behavior of the new kinds of steel. 

To date, a large number of studies involved various wear testing methods have shown that the novel nanobainitic steels possess excellent wear resistance when compared with other microstructures, such as pearlite [10,11], martensite [11,12,13,14], lower bainite [15], and so on. The carbide-free bainitic microstructure is demonstrated to exhibit a wear resistance comparable to conventional rail steels with pearlitic microstructure in a rolling/sliding wear test [10]. While the result of [11] showed that the nanobainitic microstructure presented better wear resistance in comparison to the pearlitic and martensitic microstructure through a three-body abrasive wear test. In conditions of similar hardness, the wear resistance of carbide-free bainite could enhance 25% compared to the tempered martensite [12]. Besides, for carbide-free bainite with lower hardness, the erosive-abrasive wear rate of which was also proved to be 35% and 45% lower than that of martensite [13]. Moreover, it was claimed that the specific wear rate of nanobainite was as little as 1% of that of conventional 100Cr6 with lower bainite, and approximately half of that of 100Cr6 austempered at 300 °C for 1 h [15].

The microstructural features of naonbainitic steels also had a critical influence on wear behavior. The wear resistance would be enhanced as austempering temperature decreased due to the refined lath thickness of nanobainitic ferrite [8,16,17]. In addition to bainitic ferrite size, retained austenite characteristics (including its metastability, morphology and volume fraction) is another important factor affecting the wear resistance. Film-like retained austenite is more desirable for wear behavior because it generates smaller strain-induced martensite platelets, while block-like ones with less stability transformed to coarse fresh martensite, which is more vulnerable to crack initiation and propagation [18]. However, the authors of Reference [19] thought that metastable austenite was more prone to mechanically-induced transformation owing to lower carbon content and then resulted in more hardness increment and less wear rate than nanobainite. Furthermore, it is said that a higher retained austenite content in carbide-free bainitic steel seemed to cause a worse wear performance [20]. But the case is opposite in Reference [14], which showed that a higher amount of retained austenite would improve the wear resistance of the nanobainitic steel. Hence, there is still some controversies concerning the effect of the content and stability of retained austenite on the wear performance of the steel. In addition, alloying may also influence the wear resistance of the carbide-free bainitic steel. It is claimed that aluminum promoted the formation of oxide films on the worn surface and led to the improvement of wear resistance [21].

Summarily, many earlier works have been made to study the wear resistance of nanobainite compared with traditional microstructures and the effect of microstructural features on the wear behavior of nanobainitic steels. However, the influence of varying amounts of nanobainite on the wear behavior of the steel is not very clear. Therefore, the present work is aimed to obtain different amounts of nanobainite in an Al-alloyed high carbon steel through changing austempering duration, and then evaluate the wear resistance of the steel using a pin-on-disc tester under dry sliding conditions.

## 2. Materials and Methods

### 2.1. Materials Preparation

The main chemical composition (wt.%) of the Al-alloyed high carbon steel is 1.32C-0.49Si-1.46Al-0.53Mn-1.31Cr and balance Fe. The alloy was produced using a 50 kg vacuum induction furnace and cast into an ingot of 230 mm in diameter. The ingot was first homogenized at 1200 °C for 4 h and forged into bars of 80 mm in diameter, then the bars were reheated to 1200 °C for 4 h and rolled into thinner bars with a diameter of 32 mm, and controlled cooling to 700 °C lastly. In order to reduce hardness, the steel was spheroidized at 800 °C for 9 h and cooled to 650 °C slowly in the furnace. Samples used in wear testing were machined into 30 mm in diameter and sliced into 5 mm in thickness. To ensure sufficient carbon dissolved into austenite, an austenitization temperature of 880 °C was chosen. At this temperature, there is about 1.10 wt.% C dissolved into austenite and 3.23 vol.% of carbide particles is retained, which were calculated using a Thermal-Cal software 2018 in conjunction with the database TCFE10. The martensite start temperature (Ms) of the steel after austenitization at 880 °C was determined to be 132 °C by Gleeble 3500 (DSI, New York, NY, USA). Thus, the heat treatments regimes of the samples were determined as shown in Table 1. Samples with martensite were tempered at 160 °C for 2 h to eliminate the quenching stress. The effect of tempering at 160 °C on nanobainite was thought to be negligible [22].

### 2.2. Wear Tests

The surfaces of all samples before wear testing were ground to a roughness of Ra = 0.2 μm. The pin was made from commercial quenched and tempered SAE 52100 steel with a hardness of 63 HRC and manufactured to be a cylinder with Ф 5 mm × 12.7 mm and a Ra roughness of 0.2 µm.

**Figure 1:**
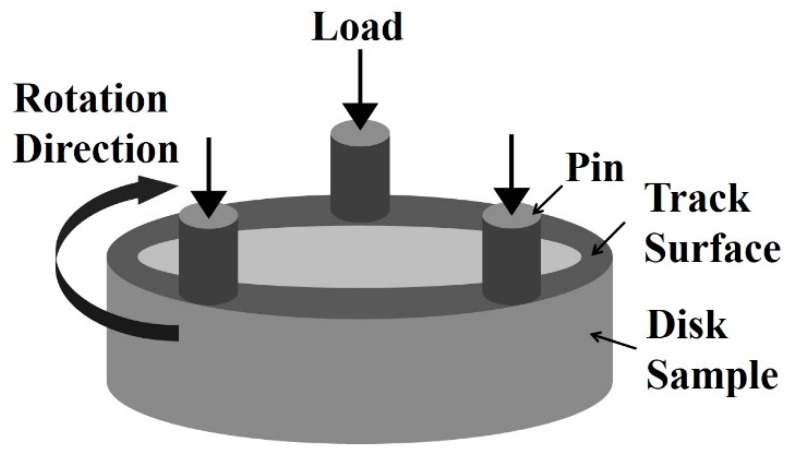
Schematic of the pin-on-disc tester.

Dry sliding tests were carried out using a home-made pin-on-disc tester, the schematic of which is shown in Figure 1. The pin was clamped to a rotating table connected to the toper shaft, the rotation speed of which was kept at a speed of 0.408 m/s. The disc sample was installed in the holder by set screw and then pressed against the disc under vertical load given by spring and kept constant through a pressure sensor. The moment of friction force between the pin and the disc sample during the wear test was measured by a moment sensor. These signals were input to a computer every second. The material pairs were self-mated and tested in ambient conditions (25–29 °C, 40–60 % humidity) without lubrication, under normal loads ranging from 25–75 N for a total sliding distance of ~8817 m. Three repetitions were carried out for each testing condition. After testing, the wear debris on worn surface was collected for X-ray diffractometry (XRD) examination, and the samples were cleaned in acetone using ultrasonic cleaner for 10 min and weighted using an electronic balance with an accuracy of 0.1 mg before and after wear test.

In order to describe the degree of wear, specific wear rate (SWR) for the studied steel with multiphase microstructures was calculated from the wear loss according to formula:

(1) $$SWR=\frac{V}{F_{N}\cdot S}=\frac{m}{F_{N}\cdot S\cdot \rho }$$

where *V* is the cumulative volume loss, *S* is the sliding distance, *F_N_* is the normal load, *m* is the total mass loss and *ρ* is the density of the steel (*ρ* = 7.85 g/cm^3^ was used in this work).

### 2.3. Microstructural Analysis

All samples used for microstructural analyses were prepared by standard procedures. After etched with 2 vol.% nital solution (2 mL HNO_3_ + 98 mL Ethanol), the microstructure was observed with optical microscope (OM, MA200, Nikon, Tokyo, Japan) and field emission scanning electron microscope (FESEM, SU8200, HITACHI UHR, Tokyo, Japan) equipped with energy dispersive X-ray spectroscopy (EDS). Transmission electron microscope (TEM, JEM-2100, JEOL, Tokyo, Japan) samples were prepared by mechanical grinding of thin foils down to a thickness of less than 50 μm followed by electrochemically thinning in a twin-jet electropolishing device, using a chemical solution of 90% CH_3_COOH and 10% HClO_4_. At least 20 TEM images were chosen to determine the ferrite true plate thicknesses *t*, by measuring the mean lineal intercept *L* = π*t*/2 in a direction normal to the plate length.

X-ray diffraction (XRD) analyses were performed on a D/MAX-2500/PC diffractometer (Rigaku, Tokyo, Japan) with unfiltered Cu Kα radiation with 40 kV and 40 mA and a scan rate of 2 °/min ranging from 35–105°. Post-wear XRD analysis was done directly on the worn surface. Quantitative analysis was used to determine the volume percentages of retained austenite in the samples before testing, using the integrated intensities of the (200)γ, (220)γ, and (311)γ peaks, together with those of (200)α, (220)α, and (211)α [23,24]. During calculation, the volume of undissolved carbides was deducted. The carbon content in the retained austenite was calculated from the lattice parameter ($a_{\gamma }$) using the Dyson and Holmes equation [25,26]:

(2) $$C_{\gamma }=\frac{a_{\gamma }-3.5780-0.00095\times {\mathrm{Mn}}_{\gamma }-0.0006\times {\mathrm{Cr}}_{\gamma }-0.0056\times \mathrm{Al}\gamma }{0.033}$$

where $a_{\gamma }$ is the austenite lattice parameter in Å and C_γ_, Mn_γ_, Cr_γ_ and Al_γ_ are the alloying concentrations in wt.%. The Mn_γ_, Cr_γ_ and Al_γ_ contents were assumed to be equivalent to the added ones in the steel. The volume percentages of nanobainite were determined by quantitative metallography on at least 10 optical images.

The hardness of worn surfaces was measured using a Vickers microhardness tester (Time, Beijing, China) with a load of 10 g and a hold time of 10 s. The impact toughness was measured using U-type Charpy impact tester (150 J, Kehui, Jinan, China).

## 3. Results and Discussion

### 3.1. Microstructure Characterization

**Figure 2:**
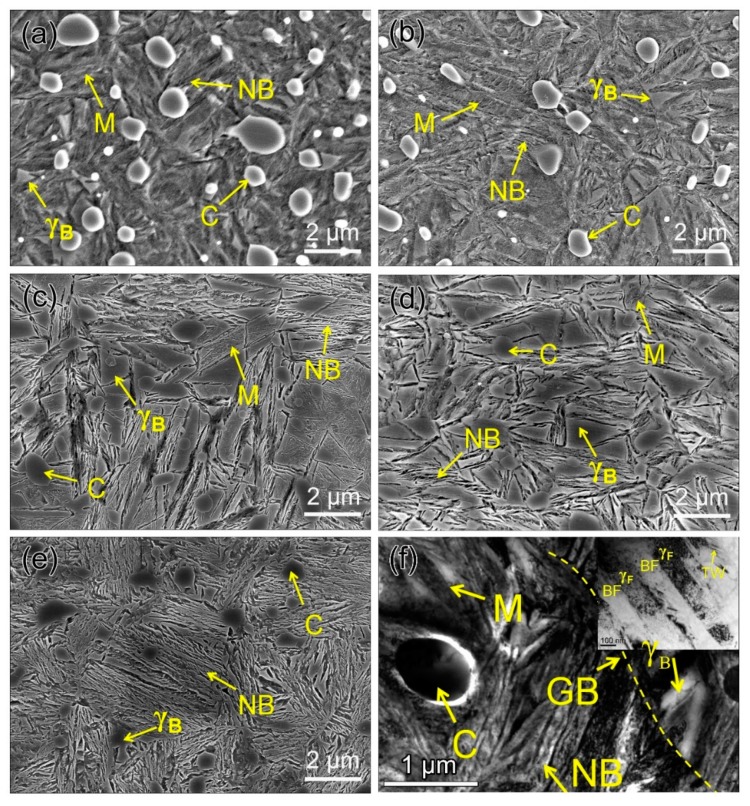
SEM images of samples used for wear testing: (**a**) B10, (**b**) B15, (**c**) B30, (**d**) B45, (**e**) B600, and (**f**) TEM images of B45 showing a mixture of NB, M, C, γ_B_ and in the corner are details of NB. NB represents nanobainite, M martensite, C carbides, GB grain boundary, γ_B_ blocky retained austenite, γ_F_ film-like retained austenite, and BF bainite ferrite, respectively.

Figure 2a–e shows the SEM images of microstructures of investigated samples after varying heat treatments. As can be seen, the amounts of nanobainite increases and that of martensite decreases as the isothermal transformation duration extends, which is presented in detail in Table 2. Meanwhile, the mean carbon content in retained austenite increases linearly with increasing austempering time owing to the increase in the amount of film-like retained austenite, which possesses higher carbon content than blocky ones. However, the total retained austenite (including γ_F_ and γ_B_) content first increases and then decreases, as the austempering duration prolongs. The microstructure details of B45 sample is characterized through TEM (Figure 2e), which shows that a multiphase microstructure composed of nanobainite, martensite, blocky retained austenite (γ_B_) and undissolved carbide was obtained. The nanobainte consists of slender bainite ferrite with 104 ± 52 nm in thickness and film-like retained austenite (γ_F_) between ferrite. In addition, accommodation twinning associated with plastic relaxation of the shape change is evident in γ_F_.

**Figure 3:**
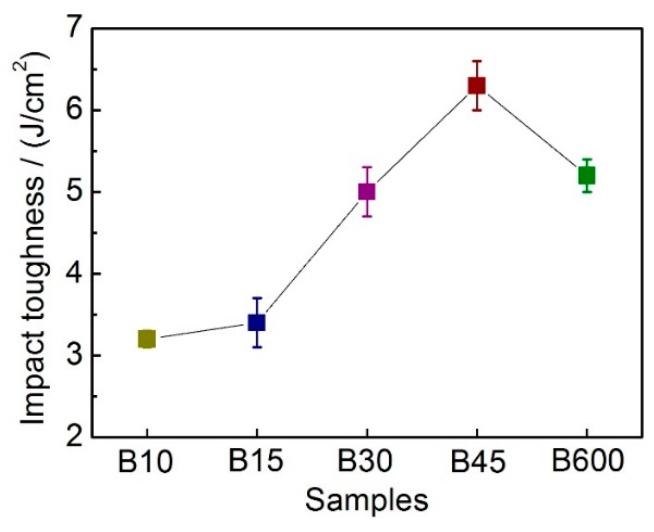
Impact toughness values of the studied steel.

Figure 3 shows the impact toughness of all studied samples. It is seen that the toughness of B10 and B15 is relatively low due to a large amount of martensite within them, which is brittle compared to retained austenite and bainitic ferrite. The optimum toughness is obtained for B45, which consists of 17.8 ± 0.3 vol.% retained austenite and 60 ± 1.8 vol.% nanobainite. When the total amount of retained austenite decreases, the toughness value decreases too, so the toughness of B600 is lower than that of B45. These results imply that the impact toughness values of the studied samples are strongly correlative with the relative quantity of nanobainite, martensite as well as γ_B_.

### 3.2. Wear Behavior

**Figure 4:**
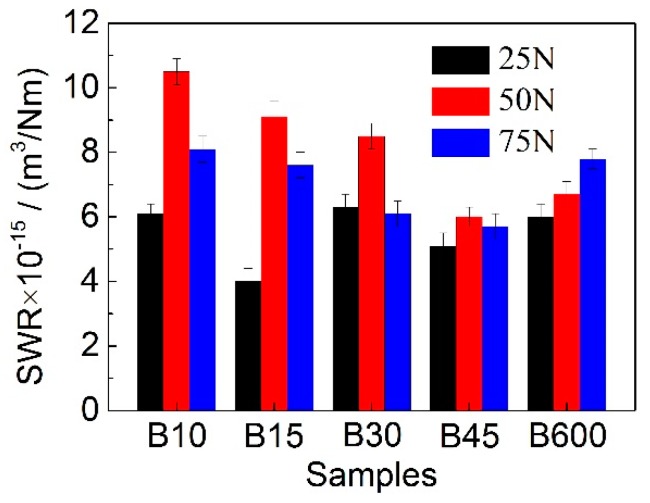
Variation of SWR of samples after wear testing under various loads.

Figure 4 shows the SWR of the studied steel with varying amount of nanobainite under diverse loads. It can be clearly observed that, the SWR gradually decreases as the nanobainite content in samples increases when the applied loads are 50 N and 75 N. The optimum wear resistance is achieved for B45 that contains a multiphase microstructure of martensite, nanobainite and γ_B_. It is claimed that microstructure with a combination of hard and soft phases is thought to be very beneficial in abrasive applications [18]. However, there is no much difference in SWR for samples tested under 25 N In addition, it is interesting that the SWR firstly increases and then decreases with increasing the applied loads for B10, B15, B30, and B45. The decrease in SWR under 75 N indicates that the multiphase microstructure may undergo a faster hardening rate than that under 50 N. Furthermore, though the quantity of nanobainite in B600 (see Table 2) is the largest, the wear resistance of which is not the best. The reason may be that the hardness of the worn surface of B600 is lower than that of B45.

### 3.3. Morphologies of Worn Surfaces and Cross-Section

**Figure 5:**
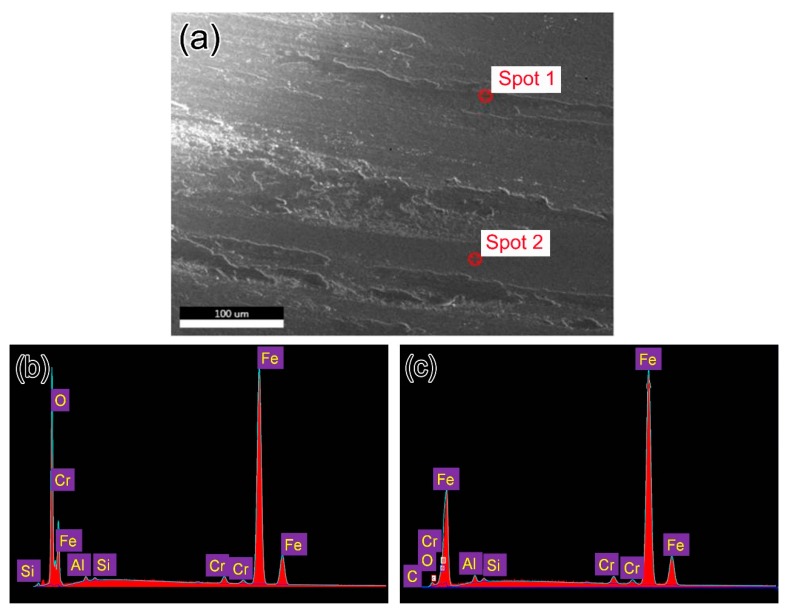
(**a**) SEM images of a typical worn surface of B45 after sliding test under 75 N, (**b**) and (**c**) EDS results corresponding to spot 1 (**b**) and spot 2 (**c**) in (**a**).

The typical SEM morphologies of worn surfaces for all studied samples is similar, as is shown in Figure 5a. It can be seen that the surface was ground to be smooth together with much debris. The chemical compositions of the wear debris and smooth region were determined by EDS, which demonstrates that the debris is mainly consists of oxygen and iron (Figure 5b) and the smooth region is matrix of the steel (Figure 5c).

**Figure 6:**
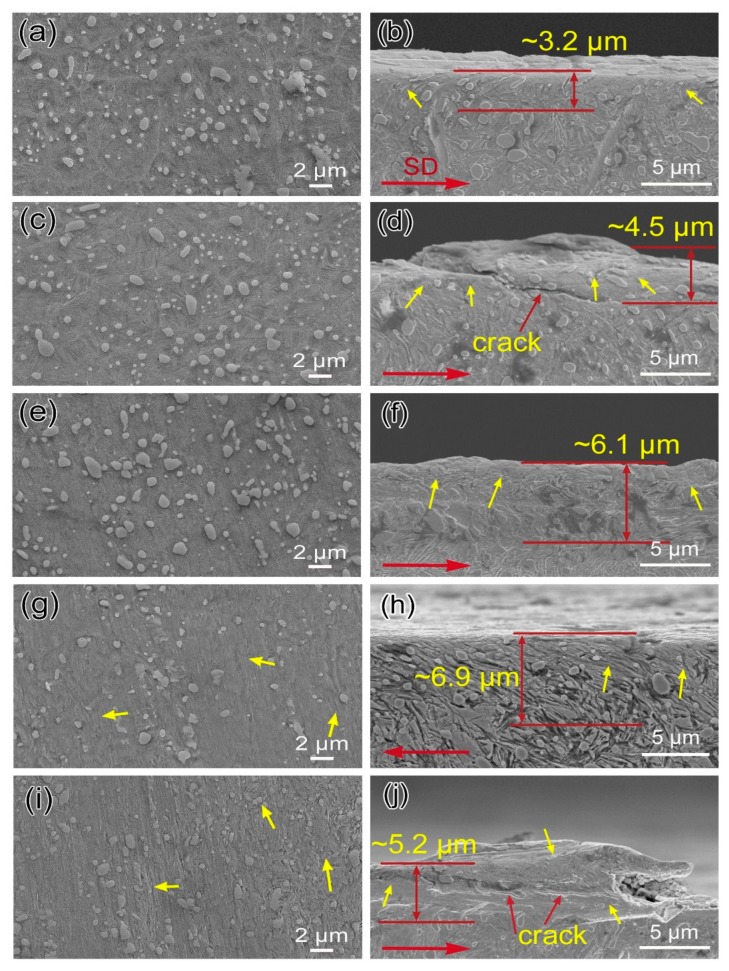
SEM images of typical worn surfaces and corresponding cross-section morphology for of B10 (**a**,**b**), B15 (**c**,**d**), B30 (**e**,**f**), B45 (**g**,**h**), B600 (**i**,**j**), under 75 N loads. Yellow arrows indicate severe plastic deformation microstructure, red parallel lines mark the approximate deformed zone, and SD indicates sliding direction.

Figure 6 shows the SEM images of directly polished and etched worn surface and the corresponding cross-section microstructure of all samples tested under 75 N. It is noted that the sliding wear results in microstructure change at the surface and subsurface layer, which is in contrast to the matrix microstructure. Besides, it is seen that the plastically deformed layer is with several micrometers in thickness, and the texture orientation of the microstructure is parallel to the friction direction. Meanwhile, a negative correlation is found between the thickness of the deformed layer and the initial hardness of the matrix, which is similar to the result of [10,20]. For instance, the thickness of the deformed layer of B45 is about 6.9 μm, which is maximum in all samples, but the matrix hardness of B45 in as-treated state is lowest. Additionally, it is noted that the initial multiphase microstructure has transformed to a single deformed microstructure plus undissolved carbide at the top-most layer, as is presented in Figure 6a,c,e,g,i. Some cracks are also seen under the hardened regions of the worn surface, as is shown in Figure 6d,j.

**Figure 7:**
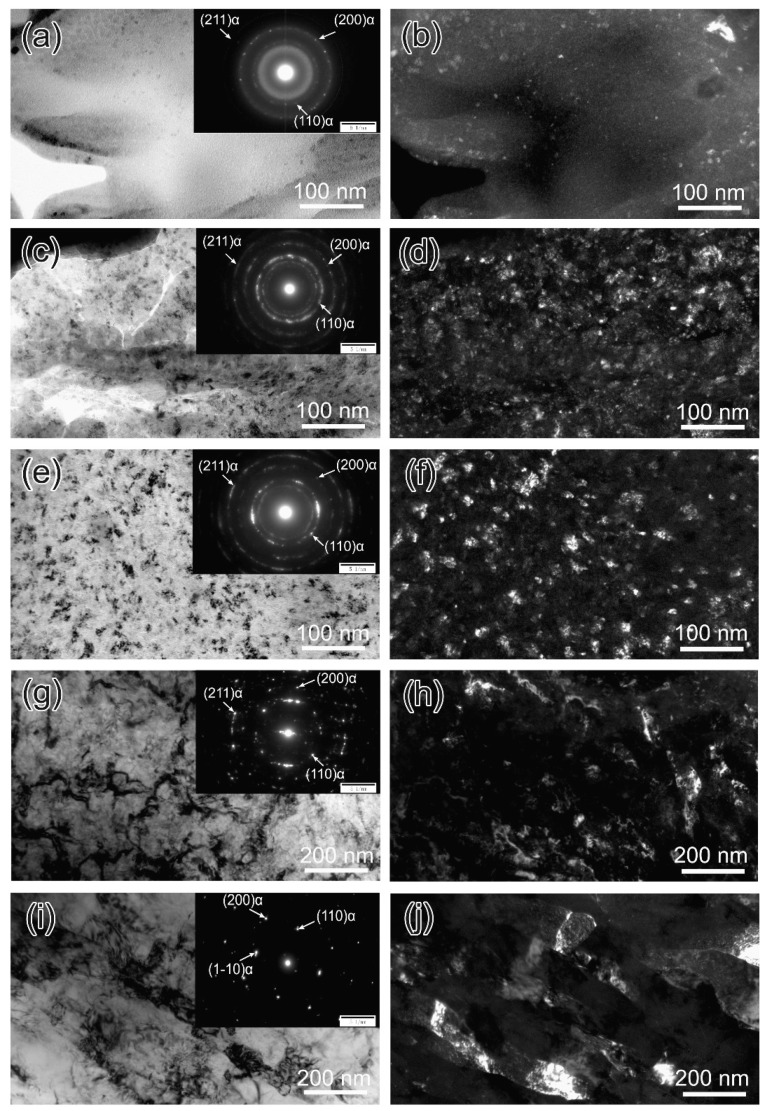
Bright (**a**,**c**,**e**,**g**,**i**) and dark (**b**,**d**,**f**,**h**,**j**) field TEM images taken from different depth of the deformed layer from the outmost surface into deeper for B45 after wear testing under 75 N, in the corner of bright field images were selected area diffraction patterns (SADPs), the surface amorphization and nanocrystallization were confirmed.

Typical TEM images of the deformed layer beneath the worn surface of B45 indicates a gradient structure formed during sliding wear process, as is shown in Figure 7. The extent of deformation gradually decreases with increasing depth of the deformed layer, and the grain size of the deformed microstructure gradually increases from 6.3 ± 2.8 nm to 160.9 ± 46.9 nm. In addition, it is clearly seen that the microstructure at the top-most layer is almost amorphous due to severe deformation at the contact surface. It is said that the grain refinement process will substantially increase the hardness and lead to a reduction of the coefficient of friction (COF), resulting in the improved wear resistance [27]. The selected-area diffraction patterns in the insets of the bright-filed micrographs show that the phase of these nano- and micro- grains is a body-centered cubic structure, which indicates that a transformation from retained austenite to martensite occurred in the deformed layer.

### 3.4. Discussion

#### 3.4.1. Effect of Hardness on Wear Resistance

**Figure 8:**
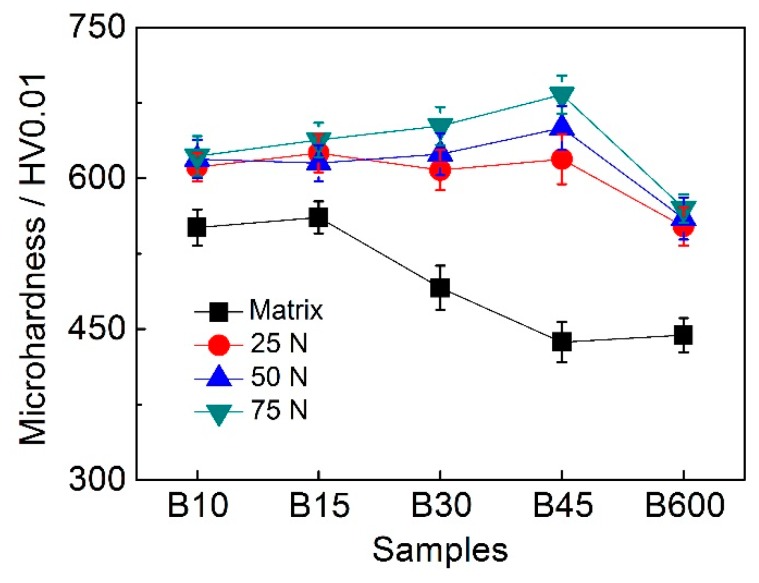
Microhardness of the matrix and worn surfaces of all samples after wear testing under 25 N, 50 N, and 75 N, respectively.

As is well known, the hardness of the material is of great importance in resisting the action of sliding forces during wear testing. Some researchers [28] claimed that wear resistance is determined by the initial hardness of materials. However, in the present study, the wear resistance of B45 is the optimum, but the matrix hardness of which is the lowest (see Figure 8). This probably because that friction always occurs at the contact surface, thus the surface hardness is in fact of great significance to wear resistance of the material. In Figure 8, one can see that the surface hardness of all samples suffered sliding wear has a remarkable increase, which can be attributed to the martensitic transformation from retained austenite, along with nanocrystallization and amorphization of surface microstructure. The occurring of phase transformation at the worn surface can be verified through XRD (see Table 2) and TEM results (see Figure 7). In addition, strain-hardening may also contribute partly to the hardness increase of the contact surface, although it is limited when comparing to γ → α′ transformation [12]. However, at a low applied load of 25 N, the hardness of the worn surfaces for all samples is very nearly the same, which results in a similar wear rate (Figure 4).

It is interesting that the hardness increase is different for different samples, as is presented in Figure 8. Since the resistance to shear strain of martensite is superior to ferrite and retained austenite, the deformed degree of the worn surface for B10 and B15 is little. However, there is more ferrite and retained austenite with high ductility in B45, thus the transformation from austenite to martensite is more remarkable. Therefore, the worn surface of B45 is the hardest and the wear resistance is also the optimum. On the other hand, it is worthy to note that the wear resistance of B600 is inferior to that of B45, despite there is more nanobainite in B600. The main reason is that the hardness of the worn surface of B600 is lower than that of B45, which is because that the total amount of retained austenite in B600 is only about half of that of B45. This is similar to Reference [14] that claimed that the sample austempered at 230 °C for 2 h with higher amount of retained austenite exhibits excellent wear resistance compared to sample austempered at 230 °C for 6 h.

#### 3.4.2. Effect of Toughness on Wear Resistance

Some researches [8,10,29] indicated that apart from hardness, toughness is another key factor influencing the wear property of the material. This is because higher toughness means the material can withstand more deformation without breaking. In the present work, the impact toughness increased as nanobainite volume percentage increased, and reached peak for B45, as shown in Figure 3. The increase in toughness was attributed to the larger quantity of nanobainite and blocky retained austenite in B45. High toughness makes the asperities at the contact surface difficult to be broken under pure shear stress. This can be confirmed by the fact that the thickness of the plastic deformation layer of B45 is greater than those of other samples. In contrast, one can see cracking happens beneath the worn surface for B15, which is due to that the hardened surface layer and the matrix of B15 is with low toughness. Although there is plenty of retained austenite in B10 and B15, it transformed less to martensite due to less plastic deformation occurred at the worn surface.

#### 3.4.3. Wear Mechanism

**Figure 9:**
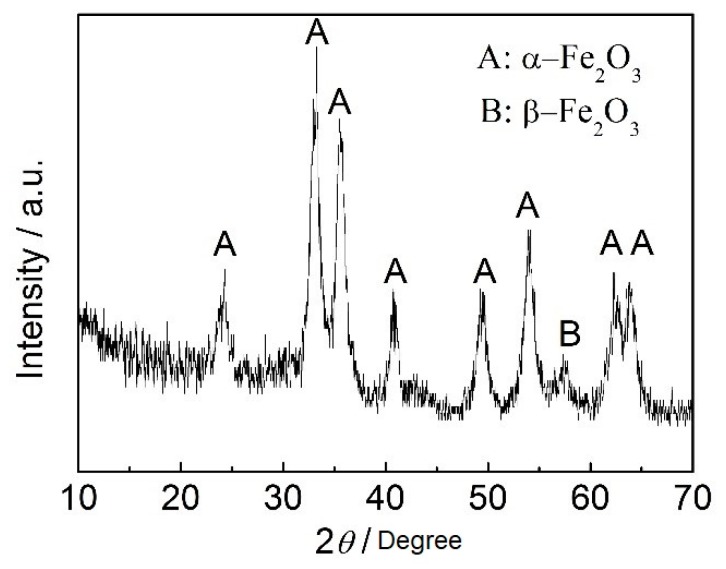
XRD pattern of the debris collected from the worn surface of B45 after wear testing under 75 N.

It is well established that, the main failure mode is oxidative wear for dry wear sliding process, during which the tribo-oxides formed due to friction heat and/or high ambient temperature [30,31]. If the applied normal load and/or the sliding speed are limited in a certain range, a continuous oxide layer will be formed by the tribo-oxides and then the wear rate can be reduced, which is called oxidative mild wear. On the contrary, if the normal load and/or the sliding speed are beyond that range, the tribo-oxides will be partly or completely removed from the wear scar and consequently lose protection to the metallic matrix, which falls into “mild-to-severe” transition region [31]. As can be seen from Figure 5a, the tribo-oxides can be identified on the worn surface, and these tribo-oxides can effectively avoid the adhesive wear between the disc samples and pin. The wear debris was verified by XRD to be Fe_2_O_3_ with α and β phase structures, as is shown in Figure 9. Therefore, the main failure mode in the present study is oxidization wear.

## 4. Conclusions

In the present work, the dry sliding wear behavior of an Al-alloyed high carbon nanobainitic steel has been studied using a pin-on-disc tester. The main conclusions are drawn as follows:

(1) A multiphase microstructure composed of nanobainite, martensite, blocky retained austenite, and undissolved carbides has been prepared in Al-alloyed high carbon steel via austempering followed by quenching heat treatments. The varying nanobainite content can be obtained through changing austempering duration.
(2) The SWR firstly decreases and then increases with the nanobainite content increases in samples. The optimum wear resistance is achieved by B45 containing 60 ± 1.8 vol.% nanobainite.
(3) The main reason for the improved wear resistance of B45 is the peak hardness increase due to deformation-induced transformation of retained austenite to martensite, work hardening, along with grain nanocrystallization and amorphization of the deformed layer. And the high toughness of the matrix is also thought to contribute to the improvement of wear resistance.
(4) The wear mechanism is proved to be oxidation wear.

## Figures and Tables

**Table 1:** Heat treatments regimes of the steel in the present study.

Sample	Austenizaiton	Austempering	Cooling	Tempering
B10	880 °C/7 min	270 °C/10 min	Cooling in oil	160 °C/120 min
B15		270 °C/15 min		
B30		270 °C/30 min		
B45		270 °C/45 min		
B600		270 °C/600 min	Ail cooled	non

**Table 2:** Volume percentages of nanobainite (*V*_NB_) and retained austenite (*V*_γ_), and the average carbon content in retained austenite (*C*_γ_) of all samples before and after wear testing (values are means ± standard deviation).

Samples	Before Wear Testing			After Wear Testing	
	*V*_NB_ (vol.%)	*V*_γ_ (vol.%)	*C*_γ_ (wt.%)	*V*_γ_ (vol.%)	*C*_γ_ (wt.%)
B10	4.2 ± 1.2	14.8 ± 0.1	1.09 ± 0.04	11.5 ± 1.8	0.92 ± 0.07
B15	9.8 ± 0.8	16.6 ± 0.5	1.10 ± 0.05	11.6 ± 1.4	0.90 ± 0.03
B30	39.4 ± 2.0	23.1 ± 2.2	1.59 ± 0.07	5.3 ± 1.2	1.05 ± 0.10
B45	60 ± 1.8	17.8 ± 0.3	1.60 ± 0.05	5.7 ± 1.2	1.31 ± 0.03
B600	~full	9.9 ± 0.1	1.91 ± 0.08	2.8 ± 0.4	-

Note: “-“denotes that the value couldn’t be obtained from XRD data.
